# A Novel Phenotype of Germline Pathogenic Variants in *MAX*: Concurrence of Pheochromocytoma and Ganglioneuroma in a Chinese Family and Literature Review

**DOI:** 10.3389/fendo.2020.00558

**Published:** 2020-08-21

**Authors:** Xiaoyan Chang, Zelin Li, Xiaosen Ma, Yunying Cui, Shuchun Chen, Anli Tong

**Affiliations:** ^1^Department of Pathology, Peking Union Medical College Hospital, Peking Union Medical College, Chinese Academy of Medical Sciences, Beijing, China; ^2^Department of Endocrinology, Key Laboratory of Endocrinology, National Health Commission of the People's Republic of China, Peking Union Medical College, Peking Union Medical College Hospital, Chinese Academy of Medical Sciences, Beijing, China; ^3^Department of Endocrinology, Hebei General Hospital, Hebei Medical University, Shijiazhuang, China

**Keywords:** *MAX*, pheochromocytoma, ganglioneuroma, adrenal, neurogenic tumor

## Abstract

**Background:** *MYC associated factor X* (*MAX*) is a tumor suppressor gene and has been identified as one of the pathogenic genes of hereditary pheochromocytoma (PCC). To date, there have been no reports of ganglioneuroma (GN) with *MAX* variants.

**Case Presentation:** The proband was a 45-years-old Chinese female with paroxysmal hypertension and palpitations who had undergone adrenalectomy for PCC 14 years ago. Her plasma free normetanephrine and 24-h urinary norepinephrine excretion were significantly increased, and abdominal computed tomography (CT) revealed an irregular mass in the left adrenal region, suggesting a recurrence of PCC. The mass was surgically removed and pathologically diagnosed as PCC with lymph node metastasis. The proband's son suffered from paroxysmal hypertension and palpitations. His plasma free metanephrine levels were normal. CT revealed a mass in the right adrenal. The tumor was surgically removed, and the pathological diagnosis was GN. Genetic testing of peripheral blood DNA revealed that the proband and her son had germline pathogenic *MAX* variant c.C97T, p.Arg33Ter, while proband's parents did not have *MAX* variants. Tumor DNA sequencing showed the same *MAX* variant (c.C97T, p.Arg33Ter) in PCC of the proband and GN of her son, both with retention of heterozygosity. Immunohistochemistry demonstrated loss of MAX protein expression in most tumor cells in PCC of the proband and some Schwannian cells in GN of the proband's son.

**Conclusion:** We report a family with a new clinical phenotype of germline pathogenic variants in *MAX* who developed both PCC and GN. Germline pathogenic variants in *MAX* may contribute to the development of GN. Our findings suggest that it is not just paternally inherited *MAX* variants that can cause tumors.

## Introduction

*MAX*, a tumor suppressor gene encoding MAX protein, locates on chromosome 14q23.3. MAX protein is part of the MYC–MAX–MXD1 complex, which acts as transcription factors to regulate cell proliferation, apoptosis, and differentiation. The MAX–MYC heterodimer activates transcription, while the MAX–MAD heterodimer inhibits transcription and MYC-dependent cell transformation (1). It has been reported that *MAX* mutations are involved in the pathogenesis of hereditary pheochromocytoma (PCC)/paraganglioma (PGL), but *MAX* mutations mainly cause PCC (2). *MAX* mutations lead to the loss of MAX protein expression, which destroys the inhibition of MYC-dependent cell transformation and thus causes tumors (3).

Germline *MAX* variants have been associated with PCC/PGL and renal oncocytoma, while somatic *MAX* mutations have been linked to the formation of other tumors such as endometrioid carcinoma and colon cancer (4). Up to now, there have been no reports of ganglioneuroma (GN) with *MAX* variants. We describe a family with germline *MAX* variants who developed PCC and GN. To our knowledge, this case is the first report of GN in a patient with germline *MAX* variants, suggesting the possibility of germline pathogenic *MAX* variants contributing to the development of GN.

## Case Presentation

### Patient 1

The proband was a 45-years-old-Chinese female who was admitted to Peking Union Medical College hospital for a recurrence of left adrenal PCC. At the age of 31 years, the patient presented with paroxysmal dizziness, sweating, palpitation, and hypertension attacks with a maximal blood pressure of 200/100 mmHg. Abdominal computed tomography (CT) indicated a mass with a size of 10 cm in the left adrenal gland. The patient underwent left adrenalectomy, and the tumor was pathologically diagnosed as PCC. Her symptoms disappeared postoperatively. However, she suffered from paroxysmal palpitation and sweating at age 45. Her blood pressure was 170/100 mmHg at hypertension attacks. Then she was referred to our hospital for further examination and treatment.

**Figure 1:**
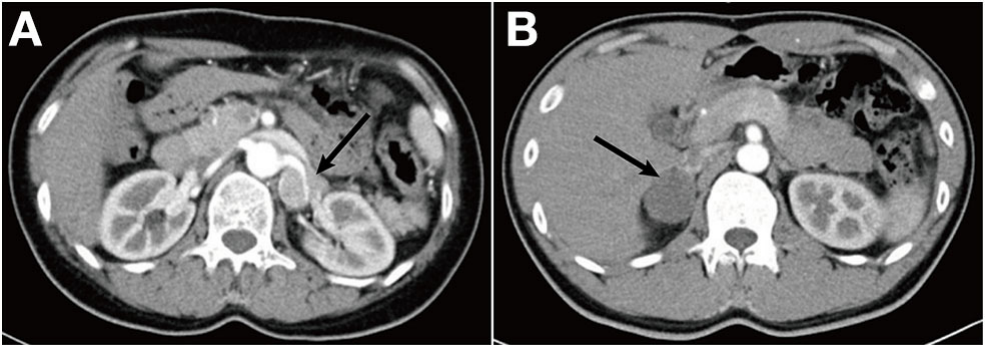
**(A)** Abdominal contrast-enhanced CT scan demonstrates an irregular left adrenal mass (4.1 × 2.7 × 3.3 cm) with a density of 42 Hounsfield unit (HU), wrapping the left renal artery. **(B)** CT scan showed a right adrenal mass (3.7 × 3.2 cm) with a density of 28 HU.

**Figure 2:**
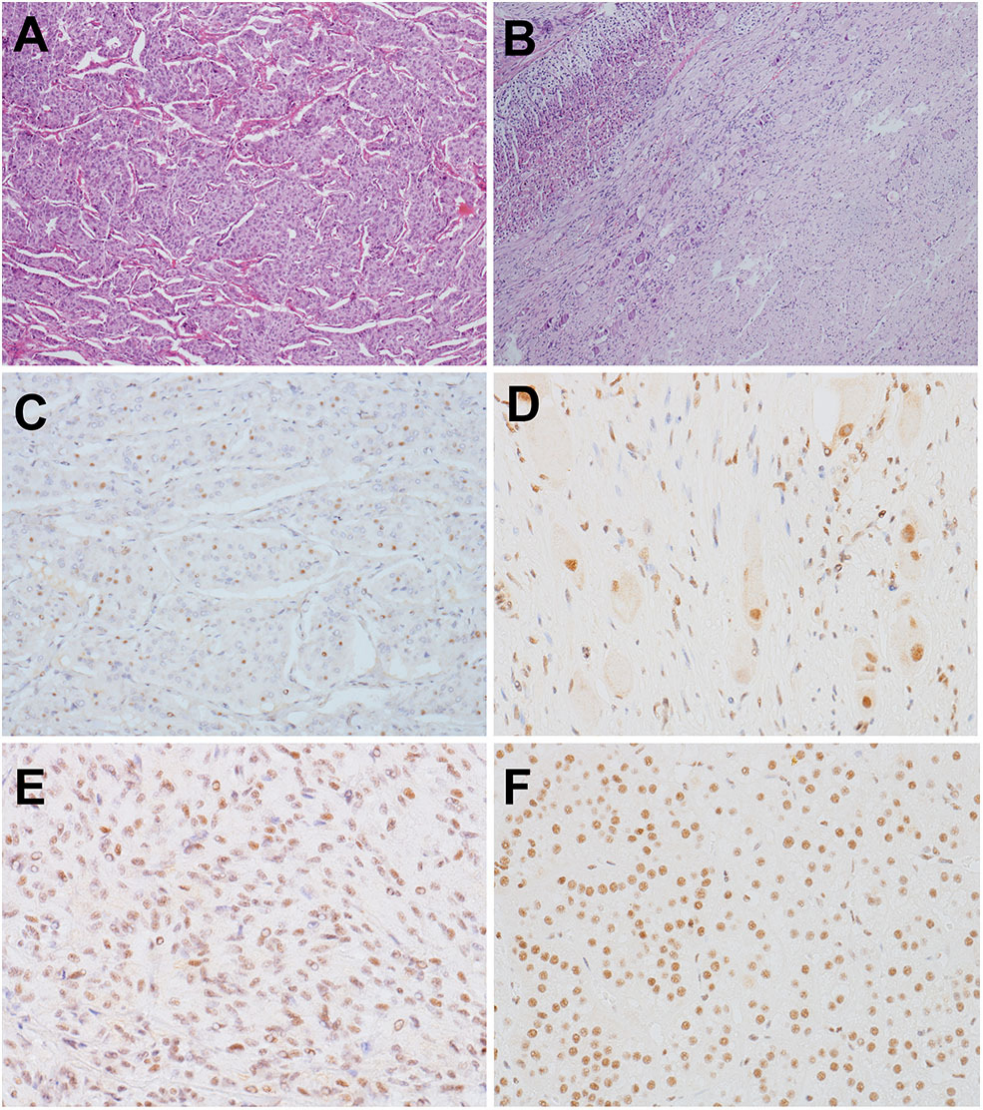
**(A,B)** HE staining (×60). **(A)** PCC of the proband. The tumor exhibited an alveolar (Zellballen) pattern. Nests of mild polygonal cells were separated by peripheral capillaries. **(B)** GN of the proband's son. The tumor located in the adrenal medulla, with a clear border to the adrenal cortex (left). The tumor was composed of scattered ganglion cells in the background of Schwannian stroma. **(C–F)** Detection of MAX by immunohistochemistry with a MAX C-terminus-specific antibody (×300). **(C)** Most tumor cells in PCC of the proband were negatively stained, but some tumor cells were positive. **(D)** The scattered ganglion cells and some Schwannian cells in GN of the son of the proband showed positive staining for MAX, but other Schwannian cells were negatively stained. **(E)** Positive staining of tumor cells in a RET-mutated PCC. **(F)** Positive staining of the normal adrenal cortex of the patient with GN.

Laboratory examinations were as follows: 24-h urinary norepinephrine, 325.44 μg/24 h (normal, 16.69–40.65); epinephrine, 4.07 μg/24 h (normal, 1.74–6.42); and dopamine, 235 μg/24 h (normal, 121–331). Contrast-enhanced CT showed multiple masses in the left adrenal, the largest of which was 2.1 × 1.3 cm, wrapping the left renal artery. It also showed multiple enlarged lymph nodes in retroperitoneum and mesenterium. Metaiodobenzylguanidine (MIBG) scintigraphy revealed the abnormally increased uptake in the left adrenal gland mass. These findings indicated recurrent PCC in the left adrenal, but the patient and her family refused surgery. The mass enlarged gradually. Three years later, contrast-enhanced CT showed an irregular left adrenal mass (4.1 × 2.7 × 3.3 cm), which was markedly enhanced and wrapping the left renal artery (Figure 1A). Plasma free normetanephrine was 9.35 nmol/L (normal, <0.9), and plasma free metanephrine was 0.46 nmol/L (normal, <0.5). The mass was surgically removed and pathologically diagnosed as PCC with lymph node metastasis (Figure 2A). Her symptoms disappeared again, and biochemical indexes decreased to normal range postoperatively. The patient had undergone partial thyroidectomy for papillary carcinoma of thyroid at the age of 46 years.

### Patient 2

Patient 2, son of the proband, was admitted to our hospital with the complaint of paroxysmal palpitation, dizziness, and headache for 2 months. At the onset of symptoms, his blood pressure was 150/90 mmHg, and his heart rate was 130 bpm. His plasma free metanephrine levels were normal. Plasma free normetanephrine was 0.30–0.66 nmol/L, and plasma free metanephrine was 0.07–0.12 nmol/L. CT scan showed a mass (3.7 × 3.2 cm) in the right adrenal with the density of 28 Hounsfield unit (HU) (Figure 1B). Neither MIBG scintigraphy nor somatostatin receptor scintigraphy (SRS) indicated any uptake in the right adrenal gland mass. The mass was removed and pathologically diagnosed as GN (Figure 2B).

The proband's parents suffered from hypertension. But examinations did not find any abdominal mass. The proband had three brothers and a sister. Their abdominal ultrasound did not show any adrenal mass. In addition to a son affected by adrenal GN, the proband had two daughters. Her daughters denied any symptoms such as headache, palpitation, sweating, or paroxysmal hypertension, and abdominal ultrasound showed no mass.

**Figure 3:**
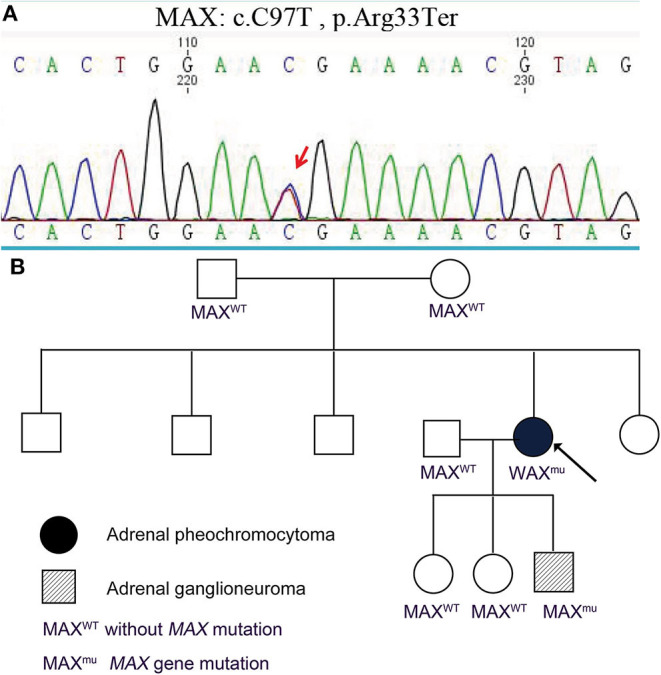
Germline pathogenic *MAX* variants in this family. **(A)** Genetic testing indicated that the proband and her son had a germline pathogenic *MAX* variant (c.C97T, p.Arg33Ter). **(B)** Pedigree of a family with PCC and GN. Other familial members including parents of the proband, her husband, and two daughters did not have *MAX* variants.

Genomic DNA was extracted from the peripheral blood of the two patients and their family members. The genomic DNA of the proband was examined for potential pathogenic germline variants of PCC by next-generation sequencing covering *SDHA, SDHB, SDHC, SDHD, SDHAF2, VHL, RET, MAX, TMEM127, FH, NF1*, and *KIF1B*. A germline *MAX* variant (c.C97T, p.Arg33Ter) was identified and confirmed by Sanger sequencing (Figure 3A). The proband's son had the same germline variant, which was detected by Sanger sequencing of the coding sequences of exon 3 of *MAX*. Other familial members including parents of the proband, her husband, and two daughters did not have *MAX* variants (Figure 3B). The minor allele frequency (MAF) of the *MAX* variant (c.C97T, p.Arg33Ter) is not specified. Mutation Taster, the Combined Annotation-Dependent Depletion (CADD), and the American College of Medical Genetics and Genomics (ACMG) predict that this variant is pathogenic.

This study was conducted with informed consent obtained from the patients and approval from the medical ethics committee of the hospital.

**Figure 4:**
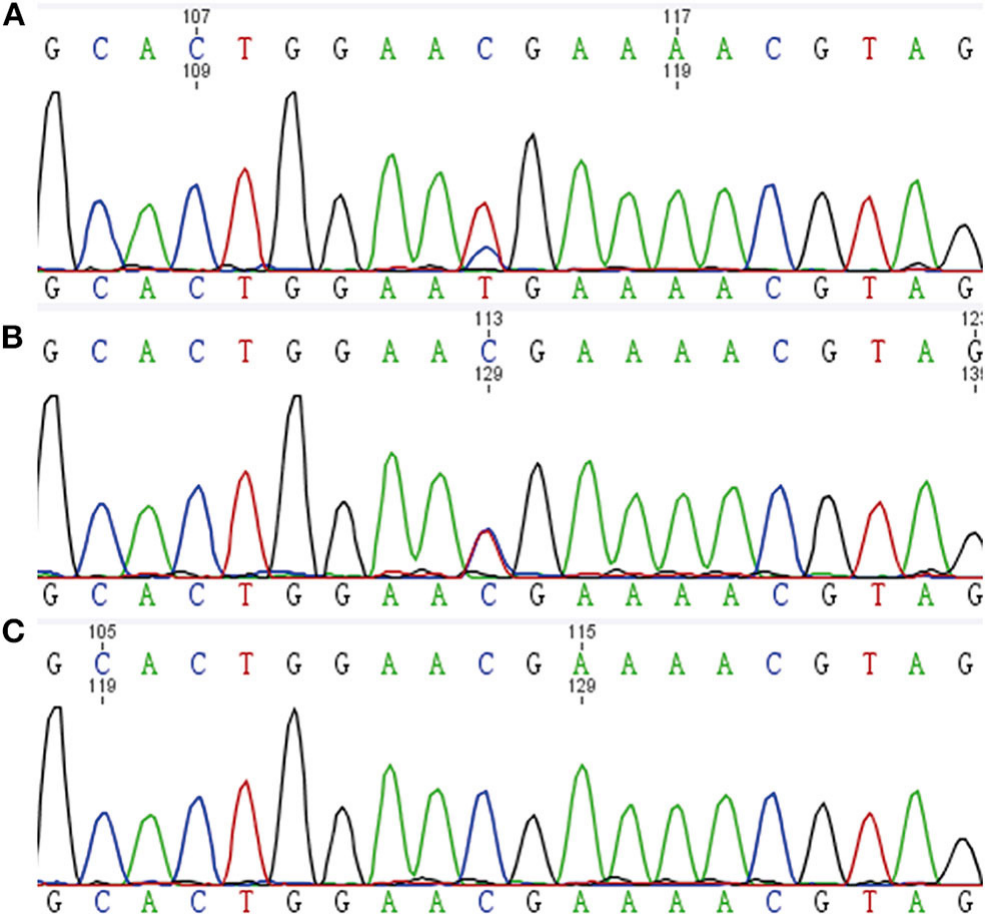
Tumor DNA sequence chromatograms. **(A)** PCC of the proband. **(B)** GN of the proband's son. **(C)** RET-mutated PCC.

DNA extracted from frozen tumor tissues of the proband and her son underwent *MAX* mutation analysis. A *RET*-mutant PCC was taken as a control. Genetic analysis of tumor DNA demonstrated that there was a *MAX* variant (c.C97T, p.Arg33Ter) in PCC of the proband and GN of her son, both with retention of heterozygosity (Figure 4).

Immunohistochemical detection was performed using the EnVision detection kit (Dako). MAX C-terminus-specific antibody was purchased from Abcam (ab101271), and the dilution was 1:1,000. Immunohistochemical staining was performed using 3-μm formalin-fixed paraffin-embedded tumor sections from the proband's PCC and her son's GN, which carry *MAX* variants. Normal adrenal cortex around GN and a PCC carrying *RET* mutations was used as controls. Only cases showing nuclear staining were considered as valuable. Immunohistochemical detection of MAX showed that most tumor cells in PCC of the proband were negatively stained, but some tumor cells were positive. And the scattered ganglion cells and some Schwannian cells in GN of proband's son showed positive staining for MAX, but other Schwannian cells were negatively stained (Figures 2C–F).

## Discussion

We describe a germline pathogenic *MAX* variant family in which the proband had PCC and her son had an adrenal GN. Neither germline *MAX*-mutated GN nor somatic *MAX*-mutated GN has been reported. To our knowledge, this is the first report of adrenal GN in a patient with germline pathogenic *MAX* variants.

PCC is a neuroendocrine tumor of the adrenal medulla, characterized by the production of excessive catecholamines, which can cause a series of symptoms such as hypertension, dizziness, palpitation, and sweating. Approximately 40% of PCCs/PGLs are caused by the germline pathogenic variants in one of almost 20 susceptibility genes (5). These susceptible genes can be classified into two major clusters according to their gene expression profile: pseudohypoxia gene cluster and the kinase signal cluster. Pseudohypoxia gene cluster includes *VHL, SDHx, HIF2A*, etc. Pathogenic variants in these genes can activate hypoxia-inducible factors and then increases the expression of hypoxia-related growth factors. Kinase signaling cluster includes *MAX, NF1, RET*, and *TMEM127*, etc. In this cluster, pathogenic variants in these genes associate with abnormal activation of kinase signaling pathways such as PI3Kinase/AKT and the mTOR pathway (6). In 2011, *MAX* was identified as a new susceptibility gene of PCC (2). Burnichon et al. reported 19 *MAX*-mutated cases in 1,694 PCC/PGL patients (1.12%), and all *MAX*-related tumors were adrenal PCCs (3). Till now, a total of ~40 patients have been reported. The *MAX* variant c.C97T, p.Arg33Ter detected in this article had been reported previously. It introduces a premature stop codon (7), thus losing the transcriptional repression of MAX protein on MYC-dependent cell transformation. *MAX* mutations are associated with bilateral PCCs, and about 10% of *MAX*-mutated patients present with metastases.

In the present case, we found a same heterozygous pathogenic *MAX* variant (c.C97T, p.Arg33Ter) in the peripheral blood and tumor tissues of both PCC and GN. The tumor DNA sequencing of PCC showed a retention of heterozygosity, which indicated that there still existed a wild-type allele. It is consistent with some tumor cells in PCC which were positive for MAX immunostaining. Nevertheless, immunohistochemistry demonstrated the loss of MAX protein expression in most PCC tumor cells, suggesting a causative role of *MAX* variants for PCC. In the previously reported cases, *MAX*-mutated tumors were most often caused by loss of heterozygosity (LOH), which was caused by either chromosomal loss or uniparental disomy and was thought to be a paternal transmission of *MAX* variants (2). However, Romanet et al. found that there still existed a wild-type allele in the *MAX*-mutated PCC. And they thought that for *MAX*-mutated tumors, in addition to chromosomal loss and uniparental disomy, other potential mechanisms can account for the loss of MAX protein expression (8). In the present case, we found that the parents of the proband did not have *MAX* variants, which suggested that the germline *MAX* variants of the proband were *de novo*. Our results are inconsistent with the loss of MAX proteins caused by the LOH related with paternal transmission of *MAX* variants. For this inconsistent result, we propose a hypothesis that there probably exist other mechanisms that cause PCC in patients with germline pathogenic *MAX* variants. LOH may occur in the development of the tumor rather than from the beginning.

GN is a rare sympathetic nervous system tumor; it occurs anywhere along the sympathetic nerve chain, about 20% of which occur in the adrenal gland (9). The pathogenesis of GN is still unclear. Most GNs are primary, but it has been reported that some GNs also arise after treatment for neuroblastoma or as a result of spontaneous neuroblastoma differentiation (10). GNs, just like PCCs, originate from neural crest cells in the neuroectoderm. In our study, there was a heterozygous pathogenic *MAX* variant (c.C97T, p.Arg33Ter) in both the peripheral blood and tumor tissues in the GN. And the scattered ganglion cells and some Schwannian cells in GN showed positive staining for MAX, but other Schwannian cells were negatively stained. These findings suggest that the pathogenic *MAX* variants may be involved in the development of GNs.

There are several reports of GN pedigrees with pathogenic variants in *RET, NF1*, and *SDHB*. *RET, NF1*, and *SDHB* genes have been implicated as causes of familial PCCs. *NF1*-mutated GNs occurred mainly in the neck, mediastinum, and retroperitoneum (11–14). *NF1* is a tumor suppressor gene, encoding neurofibromin. Loss of neurofibromin may induce abnormal proliferation of Schwann cells and PCC cells, thus causing a composite PCC–GN (15). And two studies found that *RET* variants can cause adrenal GNs in transgenic mice (16, 17). Besides *RET*-mutated adrenal GN, composite PCC–GN patients have been reported (8, 18). The *RET* gene encodes a tyrosine kinase receptor, which regulates intracellular signaling in neural crest-derived tissues. *RET* mutations could lead to neural crest-derived tumors, including PCC and GN. In addition, Niemeijer et al. reported an abdominal GN with *SDHB* negative immunoreaction, and the GN showed retention of heterozygosity (ROH) (19). These cases suggest that pathogenic variants in one of the PCC/PGL susceptibility genes can cause the GN.

Dysregulation of the MAX–MYC pathway can lead to the development of many tumors, including neuroblastoma (3). Approximately 20% of neuroblastomas have a deletion of chromosome 14q23–q32 (20), and the *MAX* gene located on chromosome 14q23.3. *MAX* may be the target of chromosome 14q loss. However, two studies did not find any *MAX* mutations in a total of 327 neuroblastoma patients (21, 22). Whether *MAX* mutations can cause neuroblastoma is still uncertain. Neuroblastoma, ganglioneuroblastoma, and GN originated from primitive sympathogonia and are collectively known as neuroblastic tumors. Neuroblastoma can mature and differentiate into ganglioneuroblastoma and GN spontaneously. Wu et al. used exome sequencing and found two 3′-UTR variants in MAX in ganglioneuroblastoma, suggesting that *MAX* variants were responsible for ganglioneuroblastoma (23). We reported a GN patient with germline *MAX* variants. Based on the above, we have a hypothesis that pathogenic *MAX* variants might be associated with neuroblastoma and that neuroblastoma then differentiates into an adrenal GN spontaneously.

In conclusion, loss of the MAX protein expression would be sufficient to suggest the causative role of germline *MAX* variants for PCC and GN. But the mechanism behind this requires more research to verify. We report a family with germline pathogenic *MAX* variants who developed both PCC and GN. This family is important in that, to our knowledge, it is the first report of a GN in a patient with a germline *MAX* variant. This case suggests the possibility of germline pathogenic *MAX* variants also contributing to the development of GN in addition to PCC. Because the *MAX* variants and GN are rare entities, the case report is important as it identifies a new clinical phenotype of germline *MAX* variants.

## Data Availability Statement

The original contributions presented in the study are included in the article/supplementary material, further inquiries can be directed to the corresponding author/s.

## Ethics Statement

Written informed consent was obtained from the individuals for the publication of any potentially identifiable images or data included in this article.

## Author Contributions

XC, XM, YC, and AT managed the case. ZL drafted the manuscript. XC, ZL, YC, SC, and AT reviewed the manuscript. XC prepared histopathological results. All authors contributed to manuscript revision, read, and approved the submitted version.

## Conflict of Interest

The authors declare that the research was conducted in the absence of any commercial or financial relationships that could be construed as a potential conflict of interest.
